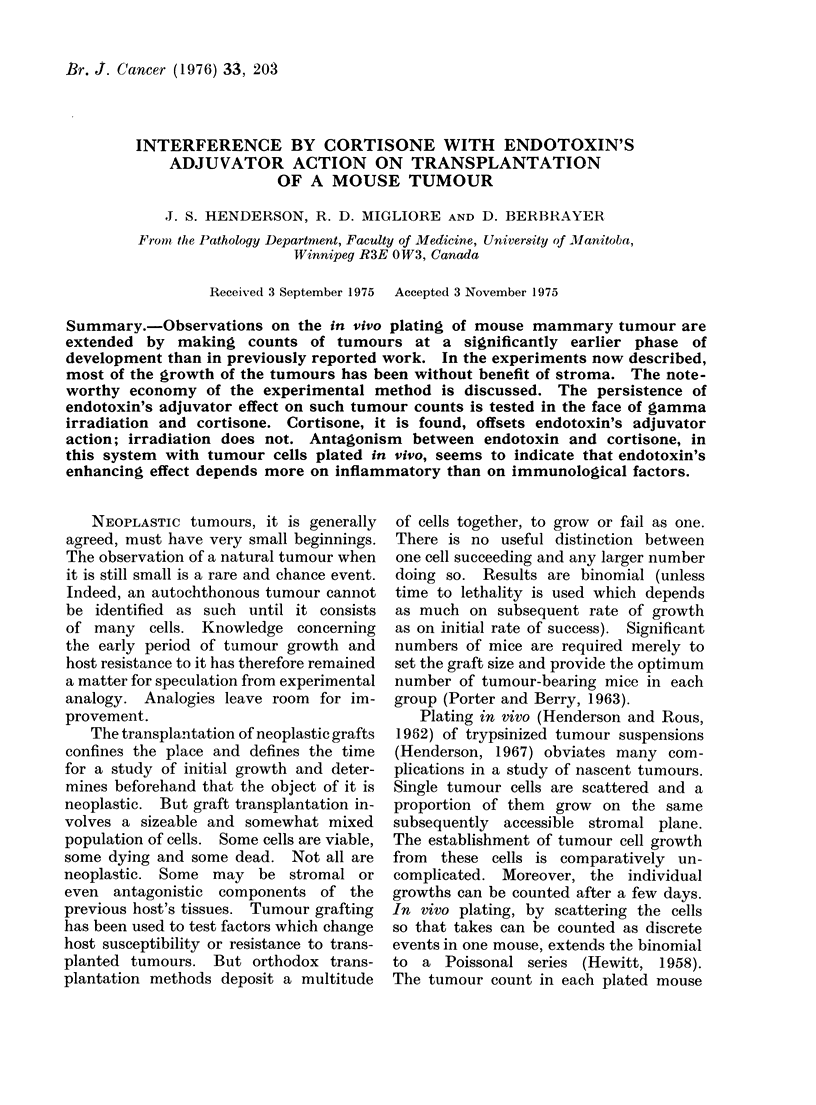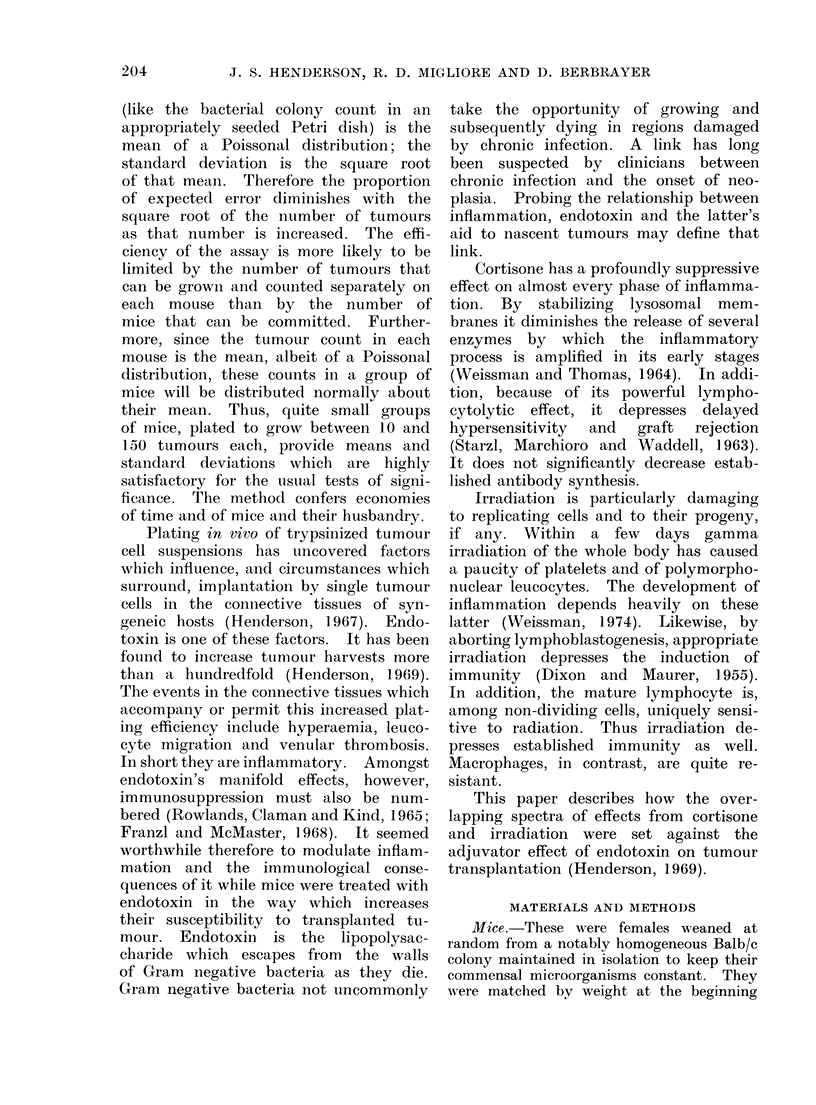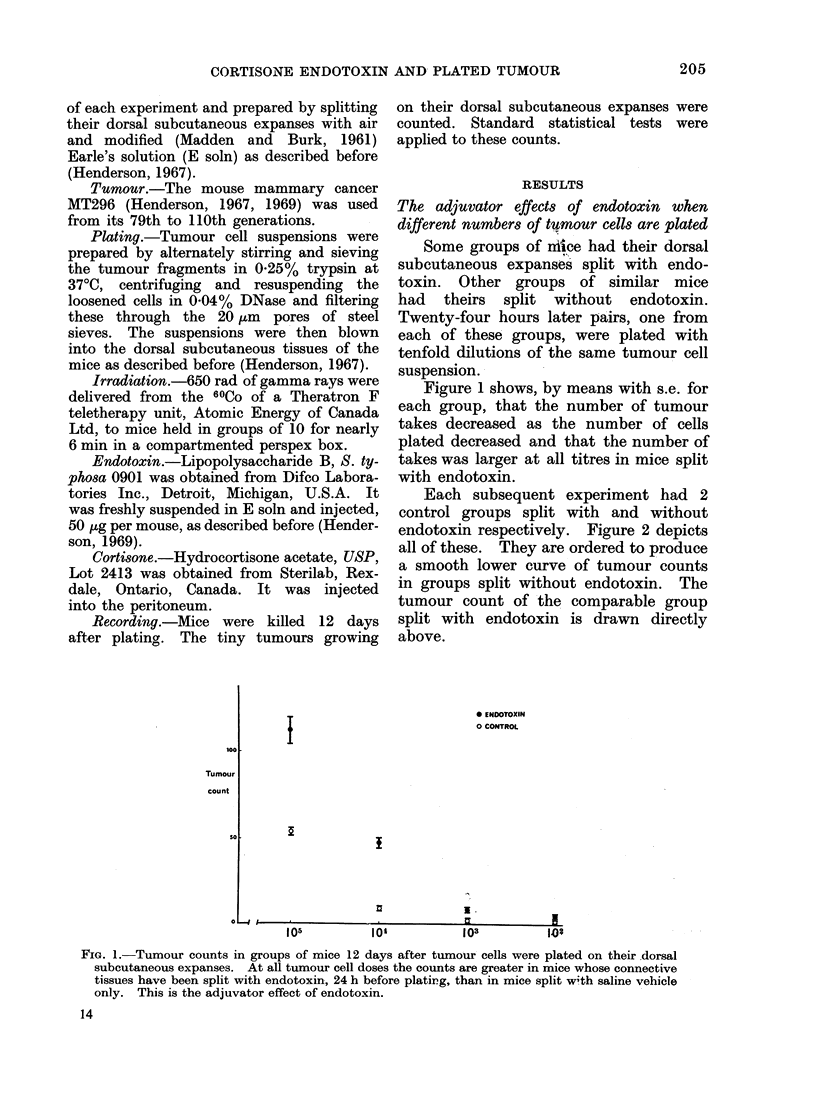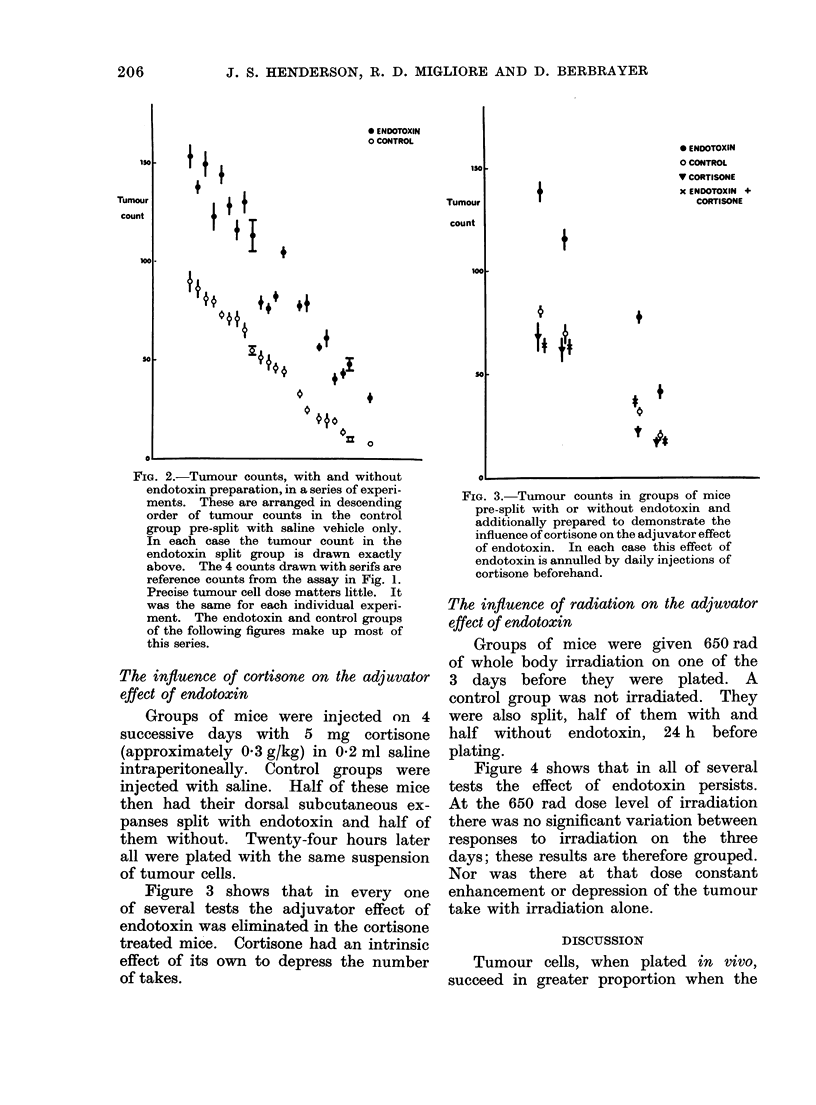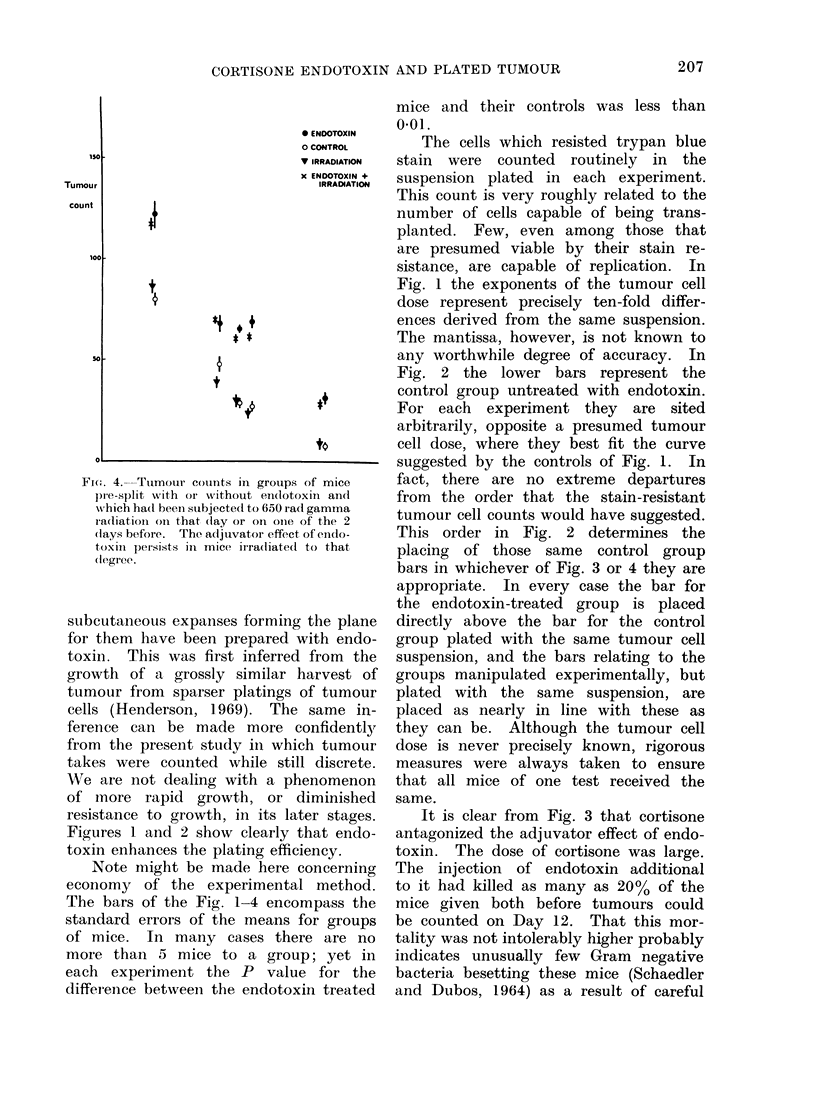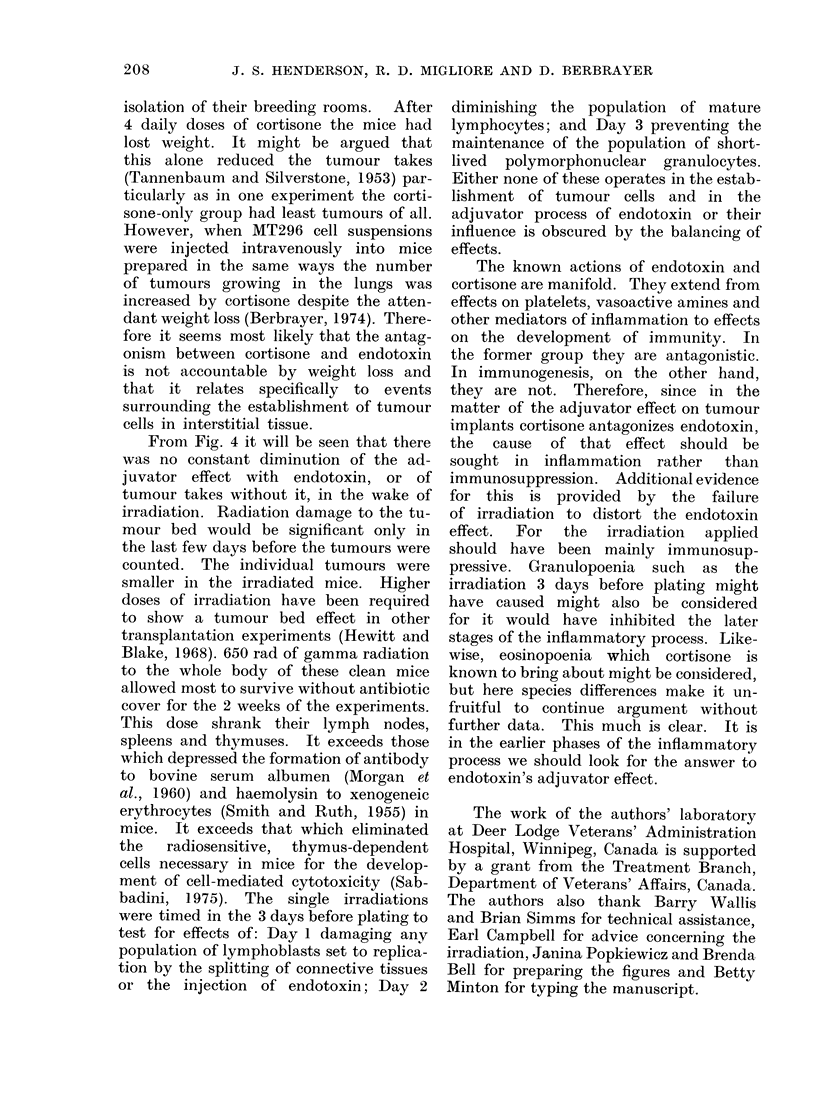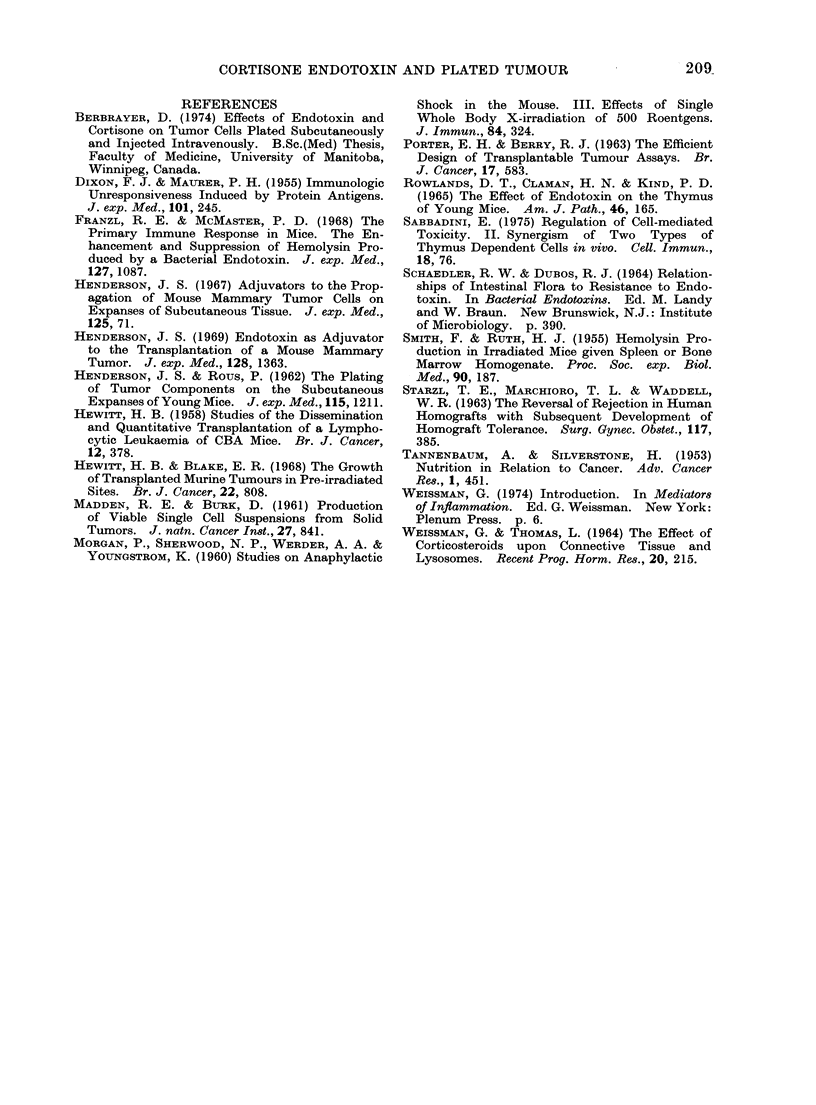# Interference by cortisone with endotoxin's adjuvator action on transplantation of a mouse tumour.

**DOI:** 10.1038/bjc.1976.26

**Published:** 1976-02

**Authors:** J. S. Henderson, R. D. Migliore, D. Berbrayer

## Abstract

Observations on the in vivo plating of mouse mammary tumour are extended by making counts of tumours at a significantly earlier phase of development than in previously reported work. In the experiments now described, most of the growth of the tumours has been without benefit of stroma. The noteworthy economy of the experimental method is discussed. The persistence of endotoxin's adjuvator effect on such tumour counts is tested in the face of gamma irradiation and cortisone. Cortisone, it is found, offsets endotoxin's adjuvator action; irradiation does not. Antagonism between endotoxin and cortisone, in this system with tumour cells plated in vivo, seems to indicate that endotoxin's enhancing effect depends more on inflammatory than on immunological factors.


*Br. J. Cancer* (1976) **33**, 203

### INTERFERENCE BY CORTISONE WITH ENDOTOXIN'S ADJUVATOR ACTION ON TRANSPLANTATION OF A MOUSE TUMOUR

J. S. HENDERSON, R. D. MIGLIORE AND D. BERBRAYER

*From the Pathology Department, Faculty of Medicine, University of Manitoba, Winnipeg R3E 0W3, Canada*

Received 3 September 1975 Accepted 3 November 1975

**Summary.—Observations on the *in vivo* plating of mouse mammary tumour are extended by making counts of tumours at a significantly earlier phase of development than in previously reported work. In the experiments now described, most of the growth of the tumours has been without benefit of stroma. The noteworthy economy of the experimental method is discussed. The persistence of endotoxin's adjuvator effect on such tumour counts is tested in the face of gamma irradiation and cortisone. Cortisone, it is found, offsets endotoxin's adjuvator action; irradiation does not. Antagonism between endotoxin and cortisone, in this system with tumour cells plated *in vivo*, seems to indicate that endotoxin's enhancing effect depends more on inflammatory than on immunological factors.**

NEOPLASTIC tumours, it is generally agreed, must have very small beginnings. The observation of a natural tumour when it is still small is a rare and chance event. Indeed, an autochthonous tumour cannot be identified as such until it consists of many cells. Knowledge concerning the early period of tumour growth and host resistance to it has therefore remained a matter for speculation from experimental analogy. Analogies leave room for improvement.

The transplantation of neoplastic grafts confines the place and defines the time for a study of initial growth and determines beforehand that the object of it is neoplastic. But graft transplantation involves a sizeable and somewhat mixed population of cells. Some cells are viable, some dying and some dead. Not all are neoplastic. Some may be stromal or even antagonistic components of the previous host's tissues. Tumour grafting has been used to test factors which change host susceptibility or resistance to transplanted tumours. But orthodox transplantation methods deposit a multitude of cells together, to grow or fail as one. There is no useful distinction between one cell succeeding and any larger number doing so. Results are binomial (unless time to lethality is used which depends as much on subsequent rate of growth as on initial rate of success). Significant numbers of mice are required merely to set the graft size and provide the optimum number of tumour-bearing mice in each group (Porter and Berry, 1963).

Plating *in vivo* (Henderson and Rous, 1962) of trypsinized tumour suspensions (Henderson, 1967) obviates many complications in a study of nascent tumours. Single tumour cells are scattered and a proportion of them grow on the same subsequently accessible stromal plane. The establishment of tumour cell growth from these cells is comparatively uncomplicated. Moreover, the individual growths can be counted after a few days. *In vivo* plating, by scattering the cells so that takes can be counted as discrete events in one mouse, extends the binomial to a Poissonal series (Hewitt, 1958). The tumour count in each plated mouse

(like the bacterial colony count in an appropriately seeded Petri dish) is the mean of a Poissonal distribution; the standard deviation is the square root of that mean. Therefore the proportion of expected error diminishes with the square root of the number of tumours as that number is increased. The efficiency of the assay is more likely to be limited by the number of tumours that can be grown and counted separately on each mouse than by the number of mice that can be committed. Furthermore, since the tumour count in each mouse is the mean, albeit of a Poissonal distribution, these counts in a group of mice will be distributed normally about their mean. Thus, quite small groups of mice, plated to grow between 10 and 150 tumours each, provide means and standard deviations which are highly satisfactory for the usual tests of significance. The method confers economies of time and of mice and their husbandry.

Plating *in vivo* of trypsinized tumour cell suspensions has uncovered factors which influence, and circumstances which surround, implantation by single tumour cells in the connective tissues of syngeneic hosts (Henderson, 1967). Endotoxin is one of these factors. It has been found to increase tumour harvests more than a hundredfold (Henderson, 1969). The events in the connective tissues which accompany or permit this increased plating efficiency include hyperaemia, leucocyte migration and venular thrombosis. In short they are inflammatory. Amongst endotoxin's manifold effects, however, immunosuppression must also be numbered (Rowlands, Claman and Kind, 1965; Franzl and McMaster, 1968). It seemed worthwhile therefore to modulate inflammation and the immunological consequences of it while mice were treated with endotoxin in the way which increases their susceptibility to transplanted tumour. Endotoxin is the lipopolysaccharide which escapes from the walls of Gram negative bacteria as they die. Gram negative bacteria not uncommonly take the opportunity of growing and subsequently dying in regions damaged by chronic infection. A link has long been suspected by clinicians between chronic infection and the onset of neoplasia. Probing the relationship between inflammation, endotoxin and the latter's aid to nascent tumours may define that link.

Cortisone has a profoundly suppressive effect on almost every phase of inflammation. By stabilizing lysosomal membranes it diminishes the release of several enzymes by which the inflammatory process is amplified in its early stages (Weissman and Thomas, 1964). In addition, because of its powerful lymphocytolytic effect, it depresses delayed hypersensitivity and graft rejection (Starzl, Marchioro and Waddell, 1963). It does not significantly decrease established antibody synthesis.

Irradiation is particularly damaging to replicating cells and to their progeny, if any. Within a few days gamma irradiation of the whole body has caused a paucity of platelets and of polymorphonuclear leucocytes. The development of inflammation depends heavily on these latter (Weissman, 1974). Likewise, by aborting lymphoblastogenesis, appropriate irradiation depresses the induction of immunity (Dixon and Maurer, 1955). In addition, the mature lymphocyte is, among non-dividing cells, uniquely sensitive to radiation. Thus irradiation depresses established immunity as well. Macrophages, in contrast, are quite resistant.

This paper describes how the overlapping spectra of effects from cortisone and irradiation were set against the adjuvator effect of endotoxin on tumour transplantation (Henderson, 1969).

### MATERIALS AND METHODS

*Mice.*—These were females weaned at random from a notably homogeneous Balb/c colony maintained in isolation to keep their commensal microorganisms constant. They were matched by weight at the beginning of each experiment and prepared by splitting their dorsal subcutaneous expanses with air and modified (Madden and Burk, 1961) Earle's solution (E soln) as described before (Henderson, 1967).

*Tumour.*—The mouse mammary cancer MT296 (Henderson, 1967, 1969) was used from its 79th to 110th generations.

*Plating.*—Tumour cell suspensions were prepared by alternately stirring and sieving the tumour fragments in 0·25% trypsin at 37°C, centrifuging and resuspending the loosened cells in 0·04% DNase and filtering these through the 20 μm pores of steel sieves. The suspensions were then blown into the dorsal subcutaneous tissues of the mice as described before (Henderson, 1967).

*Irradiation.*—650 rad of gamma rays were delivered from the $^{60}Co$ of a Theratron F teletherapy unit, Atomic Energy of Canada Ltd, to mice held in groups of 10 for nearly 6 min in a compartmented perspex box.

*Endotoxin.*—Lipopolysaccharide B, *S. typhosa* 0901 was obtained from Difco Laboratories Inc., Detroit, Michigan, U.S.A. It was freshly suspended in E soln and injected, 50 μg per mouse, as described before (Henderson, 1969).

*Cortisone.*—Hydrocortisone acetate, *USP*, Lot 2413 was obtained from Sterilab, Rexdale, Ontario, Canada. It was injected into the peritoneum.

*Recording.*—Mice were killed 12 days after plating. The tiny tumours growing on their dorsal subcutaneous expanses were counted. Standard statistical tests were applied to these counts.

### RESULTS

### *The adjuvator effects of endotoxin when different numbers of tumour cells are plated*

Some groups of mice had their dorsal subcutaneous expanses split with endotoxin. Other groups of similar mice had theirs split without endotoxin. Twenty-four hours later pairs, one from each of these groups, were plated with tenfold dilutions of the same tumour cell suspension.

Figure 1 shows, by means with s.e. for each group, that the number of tumour takes decreased as the number of cells plated decreased and that the number of takes was larger at all titres in mice split with endotoxin.

Each subsequent experiment had 2 control groups split with and without endotoxin respectively. Figure 2 depicts all of these. They are ordered to produce a smooth lower curve of tumour counts in groups split without endotoxin. The tumour count of the comparable group split with endotoxin is drawn directly above.

FIG. 1.—Tumour counts in groups of mice 12 days after tumour cells were plated on their dorsal subcutaneous expanses. At all tumour cell doses the counts are greater in mice whose connective tissues have been split with endotoxin, 24 h before plating, than in mice split with saline vehicle only. This is the adjuvator effect of endotoxin.

FIG. 2.—Tumour counts, with and without endotoxin preparation, in a series of experiments. These are arranged in descending order of tumour counts in the control group pre-split with saline vehicle only. In each case the tumour count in the endotoxin split group is drawn exactly above. The 4 counts drawn with serifs are reference counts from the assay in Fig. 1. Precise tumour cell dose matters little. It was the same for each individual experiment. The endotoxin and control groups of the following figures make up most of this series.

FIG. 3.—Tumour counts in groups of mice pre-split with or without endotoxin and additionally prepared to demonstrate the influence of cortisone on the adjuvator effect of endotoxin. In each case this effect of endotoxin is annulled by daily injections of cortisone beforehand.

*The influence of cortisone on the adjuvator effect of endotoxin*

Groups of mice were injected on 4 successive days with 5 mg cortisone (approximately 0·3 g/kg) in 0·2 ml saline intraperitoneally. Control groups were injected with saline. Half of these mice then had their dorsal subcutaneous expanses split with endotoxin and half of them without. Twenty-four hours later all were plated with the same suspension of tumour cells.

Figure 3 shows that in every one of several tests the adjuvator effect of endotoxin was eliminated in the cortisone treated mice. Cortisone had an intrinsic effect of its own to depress the number of takes.

*The influence of radiation on the adjuvator effect of endotoxin*

Groups of mice were given 650 rad of whole body irradiation on one of the 3 days before they were plated. A control group was not irradiated. They were also split, half of them with and half without endotoxin, 24 h before plating.

Figure 4 shows that in all of several tests the effect of endotoxin persists. At the 650 rad dose level of irradiation there was no significant variation between responses to irradiation on the three days; these results are therefore grouped. Nor was there at that dose constant enhancement or depression of the tumour take with irradiation alone.

DISCUSSION

Tumour cells, when plated *in vivo*, succeed in greater proportion when the

FIG. 4.—Tumour counts in groups of mice pre-split with or without endotoxin and which had been subjected to 650 rad gamma radiation on that day or on one of the 2 days before. The adjuvator effect of endotoxin persists in mice irradiated to that degree.

subcutaneous expanses forming the plane for them have been prepared with endotoxin. This was first inferred from the growth of a grossly similar harvest of tumour from sparser platings of tumour cells (Henderson, 1969). The same inference can be made more confidently from the present study in which tumour takes were counted while still discrete. We are not dealing with a phenomenon of more rapid growth, or diminished resistance to growth, in its later stages. Figures 1 and 2 show clearly that endotoxin enhances the plating efficiency.

Note might be made here concerning economy of the experimental method. The bars of the Fig. 1–4 encompass the standard errors of the means for groups of mice. In many cases there are no more than 5 mice to a group; yet in each experiment the $P$ value for the difference between the endotoxin treated mice and their controls was less than 0·01.

The cells which resisted trypan blue stain were counted routinely in the suspension plated in each experiment. This count is very roughly related to the number of cells capable of being transplanted. Few, even among those that are presumed viable by their stain resistance, are capable of replication. In Fig. 1 the exponents of the tumour cell dose represent precisely ten-fold differences derived from the same suspension. The mantissa, however, is not known to any worthwhile degree of accuracy. In Fig. 2 the lower bars represent the control group untreated with endotoxin. For each experiment they are sited arbitrarily, opposite a presumed tumour cell dose, where they best fit the curve suggested by the controls of Fig. 1. In fact, there are no extreme departures from the order that the stain-resistant tumour cell counts would have suggested. This order in Fig. 2 determines the placing of those same control group bars in whichever of Fig. 3 or 4 they are appropriate. In every case the bar for the endotoxin-treated group is placed directly above the bar for the control group plated with the same tumour cell suspension, and the bars relating to the groups manipulated experimentally, but plated with the same suspension, are placed as nearly in line with these as they can be. Although the tumour cell dose is never precisely known, rigorous measures were always taken to ensure that all mice of one test received the same.

It is clear from Fig. 3 that cortisone antagonized the adjuvator effect of endotoxin. The dose of cortisone was large. The injection of endotoxin additional to it had killed as many as 20% of the mice given both before tumours could be counted on Day 12. That this mortality was not intolerably higher probably indicates unusually few Gram negative bacteria besetting these mice (Schaedler and Dubos, 1964) as a result of careful isolation of their breeding rooms. After 4 daily doses of cortisone the mice had lost weight. It might be argued that this alone reduced the tumour takes (Tannenbaum and Silverstone, 1953) particularly as in one experiment the cortisone-only group had least tumours of all. However, when MT296 cell suspensions were injected intravenously into mice prepared in the same ways the number of tumours growing in the lungs was increased by cortisone despite the attendant weight loss (Berbrayer, 1974). Therefore it seems most likely that the antagonism between cortisone and endotoxin is not accountable by weight loss and that it relates specifically to events surrounding the establishment of tumour cells in interstitial tissue.

From Fig. 4 it will be seen that there was no constant diminution of the adjuvator effect with endotoxin, or of tumour takes without it, in the wake of irradiation. Radiation damage to the tumour bed would be significant only in the last few days before the tumours were counted. The individual tumours were smaller in the irradiated mice. Higher doses of irradiation have been required to show a tumour bed effect in other transplantation experiments (Hewitt and Blake, 1968). 650 rad of gamma radiation to the whole body of these clean mice allowed most to survive without antibiotic cover for the 2 weeks of the experiments. This dose shrank their lymph nodes, spleens and thymuses. It exceeds those which depressed the formation of antibody to bovine serum albumen (Morgan *et al.*, 1960) and haemolysin to xenogeneic erythrocytes (Smith and Ruth, 1955) in mice. It exceeds that which eliminated the radiosensitive, thymus-dependent cells necessary in mice for the development of cell-mediated cytotoxicity (Sabbadini, 1975). The single irradiations were timed in the 3 days before plating to test for effects of: Day 1 damaging any population of lymphoblasts set to replication by the splitting of connective tissues or the injection of endotoxin; Day 2 diminishing the population of mature lymphocytes; and Day 3 preventing the maintenance of the population of short-lived polymorphonuclear granulocytes. Either none of these operates in the establishment of tumour cells and in the adjuvator process of endotoxin or their influence is obscured by the balancing of effects.

The known actions of endotoxin and cortisone are manifold. They extend from effects on platelets, vasoactive amines and other mediators of inflammation to effects on the development of immunity. In the former group they are antagonistic. In immunogenesis, on the other hand, they are not. Therefore, since in the matter of the adjuvator effect on tumour implants cortisone antagonizes endotoxin, the cause of that effect should be sought in inflammation rather than immunosuppression. Additional evidence for this is provided by the failure of irradiation to distort the endotoxin effect. For the irradiation applied should have been mainly immunosuppressive. Granulopoenia such as the irradiation 3 days before plating might have caused might also be considered for it would have inhibited the later stages of the inflammatory process. Likewise, eosinopoenia which cortisone is known to bring about might be considered, but here species differences make it unfruitful to continue argument without further data. This much is clear. It is in the earlier phases of the inflammatory process we should look for the answer to endotoxin's adjuvator effect.

The work of the authors' laboratory at Deer Lodge Veterans' Administration Hospital, Winnipeg, Canada is supported by a grant from the Treatment Branch, Department of Veterans' Affairs, Canada. The authors also thank Barry Wallis and Brian Simms for technical assistance, Earl Campbell for advice concerning the irradiation, Janina Popkiewicz and Brenda Bell for preparing the figures and Betty Minton for typing the manuscript.

### REFERENCES

Berbrayer, D. (1974) Effects of Endotoxin and Cortisone on Tumor Cells Plated Subcutaneously and Injected Intravenously. B.Sc.(Med) Thesis, Faculty of Medicine, University of Manitoba, Winnipeg, Canada.

Dixon, F. J. & Maurer, P. H. (1955) Immunologic Unresponsiveness Induced by Protein Antigens. *J. exp. Med.*, **101**, 245.

Franzl, R. E. & McMaster, P. D. (1968) The Primary Immune Response in Mice. The Enhancement and Suppression of Hemolysin Produced by a Bacterial Endotoxin. *J. exp. Med.*, **127**, 1087.

Henderson, J. S. (1967) Adjuvators to the Propagation of Mouse Mammary Tumor Cells on Expanses of Subcutaneous Tissue. *J. exp. Med.*, **125**, 71.

Henderson, J. S. (1969) Endotoxin as Adjuvator to the Transplantation of a Mouse Mammary Tumor. *J. exp. Med.*, **128**, 1363.

Henderson, J. S. & Rous, P. (1962) The Plating of Tumor Components on the Subcutaneous Expanses of Young Mice. *J. exp. Med.*, **115**, 1211.

Hewitt, H. B. (1958) Studies of the Dissemination and Quantitative Transplantation of a Lymphocytic Leukaemia of CBA Mice. *Br. J. Cancer*, **12**, 378.

Hewitt, H. B. & Blake, E. R. (1968) The Growth of Transplanted Murine Tumours in Pre-irradiated Sites. *Br. J. Cancer*, **22**, 808.

Madden, R. E. & Burk, D. (1961) Production of Viable Single Cell Suspensions from Solid Tumors. *J. natn. Cancer Inst.*, **27**, 841.

Morgan, P., Sherwood, N. P., Werder, A. A. & Youngstrom, K. (1960) Studies on Anaphylactic Shock in the Mouse. III. Effects of Single Whole Body X-irradiation of 500 Roentgens. *J. Immun.*, **84**, 324.

Porter, E. H. & Berry, R. J. (1963) The Efficient Design of Transplantable Tumour Assays. *Br. J. Cancer*, **17**, 583.

Rowlands, D. T., Claman, H. N. & Kind, P. D. (1965) The Effect of Endotoxin on the Thymus of Young Mice. *Am. J. Path.*, **46**, 165.

Sabbadini, E. (1975) Regulation of Cell-mediated Toxicity. II. Synergism of Two Types of Thymus Dependent Cells *in vivo*. *Cell. Immun.*, **18**, 76.

Schaedler, R. W. & Dubos, R. J. (1964) Relationships of Intestinal Flora to Resistance to Endotoxin. In *Bacterial Endotoxins*. Ed. M. Landy and W. Braun. New Brunswick, N.J.: Institute of Microbiology. p. 390.

Smith, F. & Ruth, H. J. (1955) Hemolysin Production in Irradiated Mice given Spleen or Bone Marrow Homogenate. *Proc. Soc. exp. Biol. Med.*, **90**, 187.

Starzl, T. E., Marchioro, T. L. & Waddell, W. R. (1963) The Reversal of Rejection in Human Homografts with Subsequent Development of Homograft Tolerance. *Surg. Gynec. Obstet.*, **117**, 385.

Tannenbaum, A. & Silverstone, H. (1953) Nutrition in Relation to Cancer. *Adv. Cancer Res.*, **1**, 451.

Weissman, G. (1974) Introduction. In *Mediators of Inflammation*. Ed. G. Weissman. New York: Plenum Press. p. 6.

Weissman, G. & Thomas, L. (1964) The Effect of Corticosteroids upon Connective Tissue and Lysosomes. *Recent Prog. Horm. Res.*, **20**, 215.